# Impact of Systematic Tailored Assessment for Responding to Suicidality (STARS) Protocol Training on Mental Health Professionals' Attitudes, Perceived Capabilities, Knowledge, and Reluctance to Intervene

**DOI:** 10.3389/fpsyt.2021.827060

**Published:** 2022-02-08

**Authors:** Jacinta Hawgood, Tamara Ownsworth, Kairi Kõlves, Susan H. Spence, Ella Arensman, Diego De Leo

**Affiliations:** ^1^Australian Institute for Suicide Research and Prevention, World Health Organization Collaborating Centre for Research and Training in Suicide Prevention, School of Applied Psychology, Griffith University, Mt Gravatt, QLD, Australia; ^2^School of Applied Psychology, Menzies Health Institute of Queensland, The Hopkins Centre, Griffith University, Queensland, QLD, Australia; ^3^School of Public Health, College of Medicine and Health, University College Cork, Cork, Ireland; ^4^National Suicide Research Foundation, Cork, Ireland

**Keywords:** suicide prevention, suicide risk assessment, training evaluation, mental health professional, clinician competency

## Abstract

**Background and Aims:**

Systematic Tailored Assessment for Responding to Suicidality (STARS) protocol and associated training were developed with the key objectives of supporting clinicians to conduct a suicide enquiry, obtaining a comprehensive account of psycho-social factors contributing to suicidality, and collaboratively developing a safety plan with clients. STARS training aims to address knowledge, attitudes and capabilities that influence intervention behavior/skills. This study aimed to examine associations between clinician characteristics and pre-training competencies in suicide risk assessment (SRA), as well as the impact of STARS training workshop on clinician competencies; and to determine the predictors of SRA training outcomes.

**Method:**

Australian mental health professionals working with suicidal persons who undertook the STARS 2-day face-to-face workshop between 2018 and 2020 completed an online survey at pre- and post-training. Of the 222 participants who completed the pre-training questionnaire, 144 (64.9%) also completed the post-training questionnaire. Participants were mostly female (75.7%), had completed a university degree (86.4%), had <10 years of experience in suicide prevention (71.7%), and were allied and mental health professionals (78.1%). We used linear mixed-effects regression for statistical analyses.

**Results:**

STARS participants who reported higher perceived capability at baseline had significantly greater formal and informal training, more years of experience in suicide prevention, and were more likely to have experienced client suicide and/or suicide attempt and to report fewer SRA related fears. We found overall significant positive impacts of STARS training on clinician competencies (attitudes, perceived capability, declarative knowledge) from pre- to post-training. The most distinct changes following STARS training were for perceived capability and declarative knowledge. Participants who had more positive attitudes after training were significantly more likely to have had less prior supervision/mentoring. Reluctance to intervene was not found to significantly change after training.

**Conclusions:**

We found evidence that attitudes, perceived capability and declarative knowledge changed positively from pre- to post-STARS training among mental health professionals. Underpinned by the minimum standardized SRA competencies, STARS training may be critical for informing evidence-based knowledge and skills in SRA and safety planning.

## Introduction

Suicide is one of the leading causes of death worldwide with over 700,000 suicides recorded in 2019 (1). Comprehensive assessment and monitoring of suicidality are key to preventing suicide (2). Uncovering suicide intent and understanding the psycho-social needs of vulnerable persons require capabilities largely neglected in the training and education of mental health professionals (3, 4). The current recommendations for comprehensive suicide risk assessment (SRA) practice extend beyond administration of clinician- and client-rated risk stratification assessment tools (i.e., low-medium-high risk), which are limited by their lack of predictive reliability (5, 6). Systematic psycho-social, person-centered, needs-based assessment has been advocated as one alternative approach to SRA (7), with a focus on understanding the individual within their ecological context or social environment (6, 8).

Cramer and Kapusta (8) posited that “structured professional judgment (SPJ) of key multi-level risk factors” is needed for suicide risk assessment (p. 8). SPJ offers an opportunity to explore multiple factors guided by empirical data, to inform care decisions (9) as opposed to unstructured discretionary clinician judgement (10), which is likely to have low reliability due to its subjectivity (11). The Systematic Tailored Assessment for Responding to Suicidality (STARS) protocol (12) (see Supplementary Material 1 for protocol description) and associated training is one such SPJ approach. The STARS protocol and training were developed with the key objectives of supporting clinicians to conduct a suicide enquiry, obtain a comprehensive account of psycho-social factors contributing to suicidality, and collaboratively develop a safety plan and management response with clients (13). Clinicians' feedback on the STARS protocol highlighted the need for training to improve ease of administration and clinicians' competency and confidence with SRA (13, 14).

To date, the suicide prevention training literature is dominated by gatekeeper training (GKT), which aims to identify suicide warning signs, engage clients, and make referrals. Less emphasis has been placed on comprehensive suicide-specific training for the workforce undertaking SRA and safety planning (15, 16), and in postgraduate curriculae (17, 18). STARS training (12) is designed to enhance SRA and safety planning capabilities of the mental health workforce and addresses competencies aligned with those of Cramer et al. (15) (see Table 1 below). A 2-day training program including six modules based on these competencies was introduced in 2018, following from the original 1-day, non-mandatory STARS training [based on the original STARS protocol (2015)]. Developed by lead author JH, STARS 2-day training also includes important design input and co-facilitation by those with a lived experience of suicide. To date, has been delivered to approximately 600 mental health workers in Australia. Despite the strong uptake of training, evaluation of training impacts has lagged behind roll-out of STARS training.

**Table 1:** Suicide prevention competencies by Cramer et al. (15) and alignment with the STARS protocol training competencies.

**Competency** **[Cramer et al. (15)]**	**STARS protocol training** **Hawgood and De Leo (12)**
(1) Manage attitude and reactions toward suicide with client	•**Module 1**—Lived experience and worker attitudes •Demonstrate and describe lived experience in suicide prevention •Evaluate and apply principles supporting respectful communication/language and stigma-free documentation practice •Identify how clinician attitudes and fears influence SRA/intervention, identifying worker professional development areas
(2) Develop and maintain a collaborative, empathic stance toward client	•**Module 2**—Essential concepts in suicide risk assessment •Define critical advancements in our understanding of suicidal processes and person-centered, psycho-social approaches in SRA •Differentiate between medical (or clinician-oriented) model and person centered models of SRA
(3) Know and elicit evidence-based risk/protective factors	•Describe critical differences between warning signs and risk factors and their differing roles in SRA processes. •**Module 3**—Structure and application of STARS protocol (Parts, A, B, and C)
(4) Focus on current plan and intent of suicidal ideation	•Describe and apply the four sections of the STARS protocol, including how to record/document client and clinician observations within Part A, B, and C of the protocol •Describe and apply non-verbal and environmental measures to support rapport building and a strong therapeutic alliance •Summarize and apply essential features in psychological interview techniques as defined by Shea (19)
(5) Determine level of risk	NA—Instead of stratified risk levels, SRA formulation is based on suicidal enquiry and psycho-social needs (factors from Parts A–C)
(6) Develop and enact a collaborative evidence-based treatment plan	^a^**Module 5**—Safety planning •Describe and apply clinical elements and inter/intra-personal principles of Safety Planning according to the Stanley and Brown (20) model
(7) Notify and involve other persons	Included in Module 5 above—cont'd
(8) Document risk, plan, and reasoning for clinical decisions	•**Module 4**—Documentation and duty of care •Appropriately transcribe hypothetical case notes from Parts A, B, and C into Clinical Notes section of the STARS Protocol, preparing for Safety Planning and clinical formulation •Describe principles of Duty of Care, Reasonable Care, Consent, Foreseeability and Negligence and strategies within clinician practice in the application of the STARS Protocol that manage Duty of Care requirements
(9) Know the law concerning suicide	Included in Module 4 above—cont'd
(10) Engage in debriefing and self-care	•**Module 6**—Self care (and impacts on the worker) •Describe the potential impacts of working with suicidal persons on the worker •Define principles of self-care and identify opportunities for enhanced self-care practice related to an area of professional practice (Develop a self care plan and approach)

a *Module 5 appears before Module 4 in this column, as the STARS competencies are positioned to align directly with the Cramer et al.'s competencies adjacent to them*.

STARS training aims to address knowledge, attitudes and capabilities that influence intervention behavior/skills. Based on Bandura's (21) social cognitive theory, Burnette et al. (22) conceptual model for suicide prevention training contends that training has its effect upon clinicians' intervention behaviors by altering their competencies, as reflected by knowledge of suicide, beliefs and attitudes toward suicide prevention, reluctance or stigma, and self-efficacy to intervene. Suicide knowledge refers to both subjective and declarative understanding of suicide and its causality. Attitudes (or beliefs) toward suicide and its prevention refer to whether suicide is perceived as preventable and whether intervention with an individual who is suicidal is appropriate. Reluctance to intervene is a perception of not wanting to intervene or not being responsible for intervening to prevent suicidality. Self-efficacy refers to perceptions of capability or feeling comfortable with intervening with someone who is suicidal (22). The model also recognizes the role of individual factors in influencing the development of competencies and associated intervention behaviors including demographic (e.g., age, gender, or ethnicity) and professional characteristics (e.g., education, prior training in suicide prevention, years of experience, and discipline), as well as the role of the social context within which the practitioner intervenes (e.g., resources and organizational support for training) (22).

While originally applied to understanding impacts of GKT, this conceptualization also has utility for investigating the impact of SRA training given the overlap in areas of practice capabilities (23). Although SRA training has been shown to be associated with increased clinician competencies such as knowledge, confidence in intervention skills and attitudes toward suicide prevention (16, 24, 25) the factors influencing clinicians' response to training are important to understand so as to inform the individualization of training.

Clinicians attending SRA training vary considerably in pre-training characteristics such as job role/discipline, prior training (formal and informal), and experience in suicide prevention (26). Formal training refers to workshops or structured education in SRA or intervention, whereas informal training refers to supervision and/or mentoring for SRA practice (27). LoParo et al. (28) found that multiple sessions of formal training in SRA, as opposed to a single session, were associated with greater increases in confidence and following best-practice SRA guidelines. However, the impact of informal training or supervision on clinician competencies is under-researched, despite the recognized importance of supervision in SRA (29, 30).

In addition to the type of prior training, clinician factors such as experience of client loss to suicide or suicide attempt and fear associated with SRA and suicide related outcomes potentially influence clinician practices following training, as may their perceived capability, knowledge, and attitudes to suicide prevention. Studies have shown that 23–65% of health professionals across mental health disciplines report losing a client to suicide (26, 31). Prior experience of clients' suicide attempt or client suicide enhance fidelity to structured assessment/intervention approaches (27) and competency in SRA (31) and reduce fears of engaging in SRA and intervention (32). Conversely, lack of knowledge and confidence in SRA (33, 34), reduced self-efficacy (35), SRA related and intervention fears (36), and negative attitudes (37, 38) and beliefs (39) about suicide are associated with reduced likelihood of intervening.

The STARS protocol was developed as a comprehensive psycho-social needs-based assessment of suicidality. However, there is limited evidence concerning the outcomes of SRA training, including for STARS, as well as the influence of demographic/clinician characteristics on training outcomes. Given the potential influence of previous training and work experiences relevant to suicide prevention on SRA training outcomes, it was also of interest to examine associations between clinician characteristics (e.g., formal and informal training and loss of client to suicide) and competencies prior to training. Such findings may inform the tailoring of training to clinicians' professional development needs.

Accordingly, the current study's specific objectives are to:

- Examine associations between clinician characteristics and pre-training clinician competencies (i.e., attitudes toward suicide prevention, perceived capability in SRA, knowledge about SRA, and reluctance to intervene). It was hypothesized that greater previous formal and informal training would be associated with greater knowledge and perceived capability, more positive attitudes and reduced reluctance to intervene at pre-training.
- Determine the impact of STARS 2-day training workshop on clinician competencies. It was hypothesized that STARS training would be associated with significant improvements (from pre- to post-training) in clinician competencies such that negative attitudes to suicide prevention would decline, perceived capability would increase, knowledge about SRA would increase, and reluctance to intervene would decline.
- Determine whether clinician characteristics predict STARS training outcomes (i.e., pre-to post-training gains on clinician competencies). Following Burnette et al. (22) theory, it was hypothesized that clinician characteristics would predict greater improvements in clinician competencies.

The influence of other pre-training variables (e.g., demographics, SRA related fears, prior client suicide or attempt) on SRA training outcomes represented an exploratory component, for which there were no hypotheses.

## Method

### Procedure

Australian mental health professionals working with suicidal persons who undertook the STARS 2-day face-to-face workshop between 2018 and 2020 were invited to participate in this study. All participants were sent a link to an online survey before and after the STARS training.

All procedures were approved by the Griffith University Human Research Ethics Committee (Ref number: 2015/813/HREC). Surveys were set up in Research Electronic Data Capture (REDCap) a secure online instrument developed by Vanderbilt University to distribute surveys for research purposes (40). All participants indicated consent by proceeding with the online survey. Pre-training surveys were distributed to all registered participants up to 1 week prior to their training attendance, with three email reminders sent within this week. Post-training surveys were disseminated within 48 h post training, with three reminders up to 2 weeks post workshop.

### Survey Content and Measures

#### Predictor Variables

*Demographic factors and experiences of client suicidality*. Demographic and background information regarding age, gender, professional role, education, years in suicide prevention, SRA related fears, SRA training and supervision—more specifically, the amount of formal training (e.g., workshops) and informal training (supervision/mentoring)—and experience of client suicide and/or suicide attempt was collected. A “yes” response was allocated to participants who indicated having lost a client to suicide and/or suicide attempt, whereas a “no” was allocated to having no experiences of client suicide or suicide attempt.

*Fears about conducting SRA* included nine potential fears clinicians may face in conducting SRA. Items were informed by literature around clinician fears and anxieties concerning SRA or working with clients who are suicidal (41, 42). Specific items around fear of pushing a client toward suicide, or a client attempting or dying by suicide were based on Roush et al.'s (30) items developed specifically for their study. Participants were asked “In the past, what have been some of your reasons for not conducting a suicide risk assessment?” and were provided with a categorical list of nine common fears reported in the literature (e.g., “Fear that I might push the client toward suicide” and, “Fear of a positive answer requiring more clinical time”). Responses were rated as present or absent and the number of fears present was totalled (0–9). This scale showed good internal consistency (Cronbach's α = 0.79) for the current sample.

#### Training Outcome Variables

The STARS pre- and post-training survey includes a combination of standardized and researcher developed measures assessing clinician competency outcomes, namely, attitudes, perceived capability, declarative knowledge and reluctance to intervene.

*Attitudes to Suicide Prevention Scale* (ASP) is a 14-item scale measuring attitudes to suicide and suicide prevention and includes items associated with responsibility for suicide and its prevention (e.g., “suicide prevention is not my responsibility”), and views of suicide (e.g., “if a person survives a suicide attempt, this was a ploy for attention”) (43). A 5-point Likert scale was used, with 5 reflecting a very negative attitude and 1 reflecting a very positive attitude. An overall score (range: 14–70) was calculated by summing the scores from each item, with higher scores indicating more negative attitudes. Internal consistency for the current sample (Cronbach's α = 0.62) was lower than that reported by authors of the scale (α = 0.77) (43).

*Perceived Capability Scale* is a 5-item scale that measures a participant's perceptions of competence in SRA capabilities (12). Participants are asked to rate current level of perceived capability (e.g., “How much do you feel you know about the suicidal state?,” “…about engaging suicidal persons?,” “…about suicide risk and protective factors?”) on a 5-point Likert scale ranging from “Very little” (1) to “Everything there is to know” (5). Total scores range from 5 to 25, with higher scores reflecting greater perceived capability. This scale showed good internal consistency (Cronbach's α = 0.87) for the current sample.

*Declarative Knowledge Scale* includes 19 items directly aligned with the competencies of all training modules within the STARS training, as developed by the authors. Example items include, “Which one of the following is not a problem in current approaches to suicide risk assessment? (choose correct item)”; “Safety planning is primarily about (choose correct item)”; STARS has been founded strictly on CBT (Cognitive Behavior Therapy) models of understanding suicidality “(True or False).” Correct answers were totalled, with scores ranging from 0 to 22 (note: one item is scored out of 4; 18 items are scored 0 or 1). This scale showed good internal consistency (Cronbach's α = 0.81) for the sample.

*Reluctance to Intervene Scale* is a 9-item scale that measures reluctance to intervene with a suicidal person (44). Each item is rated on a 5-point Likert scale ranging from Strongly Disagree (1) to Strongly Agree (5). Two items are reverse-scored, and each item value is summed for a total score ranging from 9 to 45, whereby higher values mean less reluctance. This scale showed poor internal consistency (Cronbach's α = 0.58) for the current sample, although was comparable to the original testing results by the authors of the scale (α = 0.68) (44).

#### Control Variable

*Social Desirability Response Set*, a five-item survey, was included as a control variable, to assess the tendency to present oneself in an overly favorable light in terms of attitudes and behaviors (45). Given the sensitivity of the topics of inquiry and potential for demand characteristics to influence clinician's responses (46), social desirability was measured at pre-survey to inspect whether data collected on the attitudinal measures were associated with social desirability. An example item includes: “I am always courteous even to people who are disagreeable” (Item 1). Items were rated on a 5-point scale from 1 (definitely true) to 5 (definitely false), with two items reversed scored. Responses indicative of “social desirability responding” are scored as 1, while all other responses are scored 0. Total scores above 5 indicate a tendency toward socially desirable responses in answering the questionnaire. This scale showed modest internal consistency (Cronbach's α = 0.66).

### Statistical Analysis

Preliminary analyses were conducted using simple means with standard deviations, frequencies and Pearson's correlations. Further analyses used linear mixed-effect modeling to predict the main outcome measures. This method is particularly useful for repeated measures as it accounts for both within- and between-subjects variance, including the correlation between the repeated measures of participants (47). As the linear-mixed effect models expects normal distribution, we examined Q-Q plots and skewness and kurtosis; the majority of the outcome measures were within the normal distribution range [skewness or kurtosis between + 1.5 and −1.5 as by (48)]; for some, removal of extreme outliers was required.

To examine associations between baseline (pre-training) scores on the training outcome, the predictor variables of demographic and clinician characteristics including age, gender, years in suicide prevention role, experience of client suicide or suicide attempt, the amount of formal (workshops etc.) and informal training (supervision/case study), and number of SRA related fears were entered simultaneously as fixed estimates in linear mixed-effects models, in the prediction of changes in each outcome measure (attitudes, perceived capability, declarative knowledge, and reluctance to intervene). Social desirability was entered as a control variable in all analyses. To reduce multicollinearity all variables included as fixed effects were centered (49).

To measure change between pre- and post-STARS training (outcome), time was used as a categorical variable (pre vs. post) under fixed effects. In addition, the same variables as for the baseline analyses were included as fixed effects to assess their potential confounding effect in the adjusted model. Time (pre vs. post) was included as a repeated effect. First-Order Autoregressive (AR1), First-Order Autoregressive Heterogenous (ARH1) and Unstructured (UN) covariance structures were examined using −2 Res Log Likelihood and Akaike's Information Criterion (AIC). All structures were applied to the levels of group (location)^*^person (as STARS is delivered in groups, which means that participants are nested within groups). Random intercepts for participants were included to model for the correlation of within person factors at baseline. The ARH1 structure was identified as the model with the best fit with all outcome variables. Effect sizes were not computed because there is no broad agreement on which should be used with designs of this type.

To identify predictors of change in outcome measure, we measured change for all outcome measures by subtracting the post-test from the pre-test score [i.e., post minus pre; (50)]. The same variables as for the baseline analyses were included as fixed effects, and location of the workshop (coded 1–13) was entered as random effects.

A comparison was made between those who completed the survey both before and after the training with those who completed the survey only prior to the training. This showed that time working in suicide prevention was significantly different [Chi2 = 4.03 (df = 1) *p* = 0.045; further details in SM 3], with individuals working less years in suicide prevention being less likely to complete the post-training survey. Nevertheless, linear mixed-effects regression accommodates unbalanced data with the assumption that missing data are missing at random in the outcome measures (time: before, after). Cases with missing values were not dropped from the analyses, however, missing data for the predictor variables was addressed by listwise deletion of cases. We first used Little's test of missing completely at random (MCAR) and identified that there was no systematic variation between missing data points in different sets used for different outcome measures and separately also including post data. Therefore, we concluded that data was MCAR. In general, it has been shown that multiple imputation does not change the results with repeated measures (51). Over 10% of missing cases were identified due to missingness in predictor variables. Therefore, we conducted further sensitivity analyses with multiple imputation (*n* = 10) for the STARS impact. A probability level of 0.05 was employed for all statistical tests. IBM SPSS ver 27.0 was used.

## Results

### Participants

The demographic and clinical characteristics of participants are presented in Table 2. A total of 222 participants completed the pre-training survey. Participants were mostly female (75.7%), had completed a university undergraduate or post-graduate degree (86.4%), were non-Indigenous (88.7%), had <10 years of experience in suicide prevention (71.7%), and were allied and mental health professionals (78.1%). Of the 222 participants who completed the pre-training questionnaire, 144 (64.9%) also completed the post-training questionnaire. After the first STARS training workshop, the researchers considered more closely the theoretical model of training evaluation and influences on intervention behavior by Burnette et al. (22), which suggests that reluctance be measured as a variable potentially amendable to training influences. Therefore, the measure of reluctance was only incorporated into the survey administration for 12 out of 13 workshops in this study. Data were available for 189 participants on this measure at pre-training which was used for the mixed effects model analysis.

**Table 2:** Description of study participants.

		***N***	**%**
Gender	Male	44	19.8
	Female	168	75.7
	Other	10	4.5
Age group (missing = 17)	24–34	50	24.4
	35–44	70	34.1
	45–54	55	26.8
	55–64	23	11.2
	65+	7	3.4
Ethnicity (missing = 36)	Indigenous	21	11.3
	Non-indigenous	165	88.7
Work role (missing = 21)	Allied Health and MH workers	157	78.1
	Human services (e.g., welfare worker)	44	21.9
Education (missing = 2)	High school/vocational (TAFE)	30	13.6
	University undergraduate degree	89	40.5
	University postgraduate degree	101	45.9
Years in suicide prevention (missing = 10)	<10 years	152	71.7
	Equal to or More than 10 years	60	28.3
Amount of formal training	None at all	22	9.9
	Some	136	61.3
	Moderate to a lot	64	28.8
Amount of informal training (missing = 1)	None at all	29	13.1
	Some	98	44.3
	Moderate to a lot	94	42.5
Experience of client suicide or attempt (missing = 3)	No	97	44.3
	Yes	122	55.7
Number of fears	None	130	58.6
	1	51	23.0
	2	13	5.9
	3	11	5.0
	4 or more	17	7.7

### Associations Between Clinician Characteristics and Pre-training Competencies

Correlation analyses conducted between pre-training competencies and clinician characteristics (see Table 3) identified that negative attitudes to suicide prevention had significant but weak correlations with number of fears about conducting SRA (*r* = 0.16, *p* = 0.023). Perceived capability had a strong positive correlation with amount of formal training (*r* = 0.48, *p* < 0.001) and informal training (*r* = 0.47, *p* < 0.001), a moderate positive correlation with years of experience in suicide prevention (*r* = 0.34, *p* < 0.001). Further, greater perceived capability was related to having prior experience of client suicide and/or suicide attempt (*r* = 0.31, *p* < 0.001), and was associated with fewer SRA related fears (*r* = −0.30, *p* < 0.001). Likewise, more declarative knowledge was significantly (but weakly) correlated with having prior experience of client suicide or suicide attempt (*r* = 0.14, *p* = 0.002) and was moderately correlated with more informal training (*r* = 0.24, *p* < 0.001). Reluctance to intervene had significant, but weak correlation by gender (*r* = −0.17, *p* = 0.024) with females presenting lower levels of reluctance.

**Table 3:** Correlations of variables included at baseline.

	**[1]**	**[2]**	**[3]**	**[4]**	**[5]**	**[6]**	**[7]**	**[8]**	**[9]**	**[10]**	**[11]**	**[12]**
Attitudes to Suicide Prevention Questionnaire [1]	–											
Perceived Capability Scale [2]	−0.029	–										
STARS declarative knowledge [3]	−0.124	0.235^**^	–									
Reluctance [4]	−0.530^***^	−0.008	0.090	–								
Gender (male vs. female) [5]	−0.105	−0.062	0.097	0.156^*^	–							
Age [6]	0.013	0.131	−0.058	−0.114	−0.023	–						
Years in suicide prevention sector [7]	0.021	0.338^***^	0.103	−0.027	0.008	0.378^***^	–					
Amount of formal training (workshops etc.) [8]	−0.087	0.477^***^	0.126	0.092	−0.004	0.099	0.268^***^	–				
Amount of Informal training (supervision etc.) [9]	−0.097	0.472^***^	0.237^**^	0.046	0.076	0.052	0.344^***^	−0.474^***^	–			
Experience of client's suicide or suicide attempt [10]	−0.059	0.309^***^	0.136^*^	−0.062	0.029	0.166^*^	0.286^***^	0.265^***^	0.338^***^	–		
Number of fears of [11]	0.155^*^	−0.301^***^	0.081	−0.078	−0.057	−0.233^**^	−0.200^**^	−0.166^*^	−0.151^*^	−0.109	–	
Social desirability score [12]	−0.180^**^	0.010	−0.059	0.159^*^	0.138^*^	0.106	−0.041	−0.027	−0.023	−0.082	−0.208^**^	–

*** *p < 0.001;*

** *p < 0.01;*

* *p < 0.05*.

The main fixed effects for the linear mixed-effects regression for examining the effects of predictor variables upon outcome are presented in Table 4. None of the expected predictors remained significant in models predicting the baseline scores for attitudes, declarative knowledge and reluctance. However, baseline or pre-training perceived capability was predicted by amount of formal (*F* = 8.51, *p* < 0.001) and informal (*F* = 6.39, *p* = 0.002) training, whereby an increased amount of training was associated with greater perceived capability. In addition, higher perceived capability at baseline was associated with the experience of a client suicide and/or suicide attempt (*F* = 5.16, *p* = 0.024) and lower number of SRA related fears (*F* = 10.03, *p* = 0.002) predicted capability.

**Table 4:** Fixed effect estimates for the predictors of the main outcome measure at baseline (pre-training) from the linear mixed-effects models.

	**Attitudes**		**Capability**		**Declarative knowledge**		**Reluctance**	
	**(*N* = 195)**		**(*N* = 195)**		**(*N* = 195)**		**(*N* = 169)**	
	***F***	***P*-value**	***F***	***P*-value**	***F***	***P*-value**	***F***	***P*-value**
Gender	1.45	0.237	0.59	0.555	0.95	0.388	1.30	0.276
Age	0.28	0.598	0.39	0.534	1.16	0.282	2.28	0.133
Amount of formal training (workshops etc.)	0.14	0.866	8.51	<0.001	0.05	0.953	0.62	0.0542
Amount of informal training (supervision, mentoring)	0.75	0.475	6.39	0.002	2.35	0.099	0.54	0.586
Years in suicide prevention sector	1.81	0.180	3.54	0.062	1.05	0.306	0.01	0.930
Experience of client attempt or suicide	1.44	0.231	5.16	0.024	0.64	0.426	0.39	0.534
Number of different fears	1.60	0.208	10.03	0.002	1.34	0.248	0.55	0.460

*All analyses are adjusted for social desirability*.

### Change in Competencies From Pre- to Post-training

**Figure 1:**
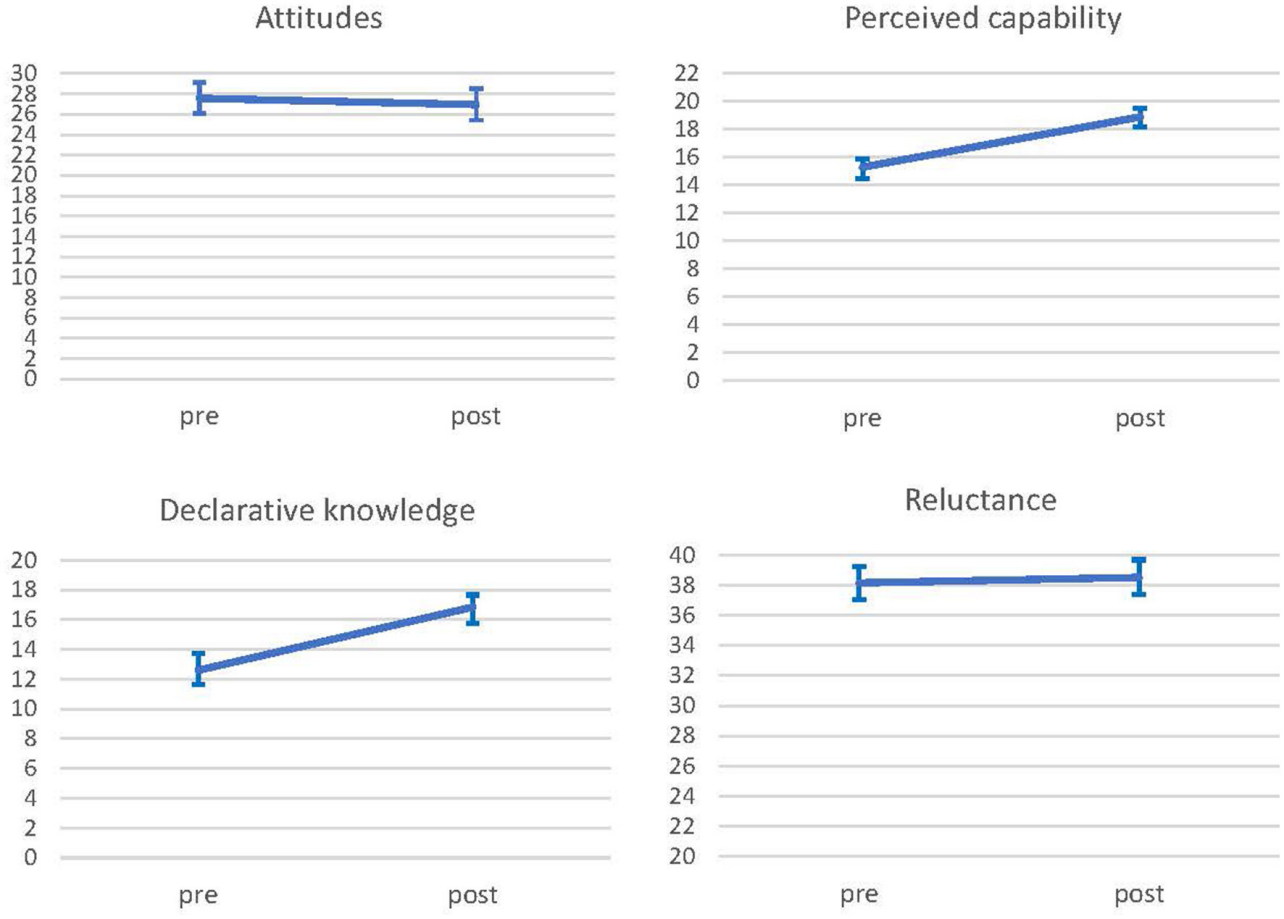
Estimated marginal means of main outcome measure pre and post intervention.

Table 5 presents the fixed estimate in the change in time (pre vs. post-training competencies) including unadjusted and adjusted models. Figure 1 shows changes for all outcome measures using estimated marginal means with their confidence interval. The results show significant changes in all main outcome measures except for reluctance. Significant increases were evident for perceived capability (*F* = 232.48, *p* < 0.001), declarative knowledge (*F* = 176.56, *p* < 0.001) and attitudes (*F* = 6.24, *p* = 0.014). Further sensitivity analyses using multiple imputation showed the same results.

**Table 5:** Fixed effect estimates of time (change pre to post) predicting main outcome measures in unadjusted and adjusted linear mixed-effects models.

	**Unadjusted**			Adjusted^*^		
	***N***	***F***	***P*-value**	***N***	***F***	***P*-value**
Attitudes	215	6.24	0.014	189	4.54	0.035
Perceived capability	219	286.98	<0.001	194	232.48	<0.001
Declarative knowledge	219	206.51	<0.001	195	176.56	<0.001
Reluctance	189	2.42	0.123	164	1.48	0.227

* *Adjusted for age, gender, years in suicide prevention role, experiencing client suicide or suicide attempt, the amount of formal training and informal training, number of fears, social desirability score*.

### Predictors of Change

The fixed-effect estimates in the models examining predictors of training outcomes (post minus pre-training scores on each outcome measure) are presented in Table 6. When all predictor variables were entered simultaneously, controlling for social desirability, change in attitudes (ASP: desired change = decline) was predicted only by amount of previous informal training (*F* = 4.42, *p* = 0.014); more specifically, those with more previous informal training (moderate to a lot) reported less attitudinal change. Change in capability (desired change = increase) was predicted only by amount of previous formal training (*F* = 5.49, *p* = 0.005); those with more previous formal training showed less improvement in capability. Change in declarative knowledge (desired change = increase) was predicted only by gender (*F* = 3.43, *p* = 0.036; those reporting their gender as “other” (*N* = 10) showed greater change in declarative knowledge compared to females (reference group), while there was no difference between males and females.

**Table 6:** Fixed effect estimates predicting change (post-pre) of main outcome measures in linear mixed-effects models.

	**Attitudes**		**Capability**		**Declarative knowledge**		**Reluctance**	
	***F***	***P*-value**	***F***	***P*-value**	***F***	***P*-value**	***F***	***P*-value**
Gender	0.61	0.547	0.15	0.864	3.43	0.036	0.27	0.761
Age	<0.01	0.969	0.97	0.326	0.95	0.331	3.67	0.058
Amount of Formal training (workshops etc.)	2.44	0.093	5.49	0.005	0.38	0.682	1.75	0.180
Amount of Informal training (supervision, mentoring)	4.42	0.014	2.25	0.110	1.12	0.330	0.07	0.932
Years in SP sector	0.21	0.650	0.80	0.374	<0.01	0.959	0.07	0.792
Experience of client's suicidality (SA or suicide)	0.66	0.418	1.53	0.218	0.17	0.685	0.30	0.582
Number of different fears about conducting SRA	2.06	0.154	3.34	0.070	0.03	0.860	<0.01	0.955

*All analyses are adjusted for social desirability*.

## Discussion

STARS is a client-centered, psycho-social, needs-based, semi-structured interview protocol (12). STARS training is designed to support acquisition of clinician competencies required for comprehensive SRA and safety planning. Understanding the impacts of STARS training on clinician competency is critical for ongoing quality assurance and evaluative purposes.

### Clinical Characteristics and Pre-training Competencies

Participants' demographic and clinical characteristics were generally not associated with the baseline clinician competency measures in the cross-sectional correlations at baseline. However, based on the final model participants who reported higher perceived capability at baseline tended to have had greater formal and informal training, more years of experience in suicide prevention, and to be more likely to have experienced client suicide and/or suicide attempt and to report fewer SRA related fears. The association between perceived capability at baseline and the amount of formal and informal training is consistent with the results of both LoParo et al. (28, 52). Also, the finding that perceived capability in SRA was associated with having prior experience of a client suicide/attempt and lower number of fears around SRA is consistent with previous studies (53, 54), and in accordance with a systematic review (55). Lund et al. (54) found that more frequent experience working with suicidal clients was related to higher perceived competency with suicide prevention. Silva et al. (53) found that health care staff who had more clinical contact, and those who experienced a client suicide, had higher confidence scores in SRA training. The current findings extend upon previous research by demonstrating that, in mental health professionals undergoing SRA training, perceptions of capability were higher at baseline for those with more training, more years of experience, greater probability of having experienced client suicide/attempt and having fewer fears about SRA practice. A possible explanation for this finding is that professionals who have higher perceived capabilities may, by virtue of their role, have more frequent contact with suicidal clients, thus increasing the likelihood of having experience of client suicide/attempt.

Due to the cross-sectional nature of the data, it is not possible to determine whether fears of engaging in SRA practice contribute to or are influenced by perceived capability. Nonetheless, the negative association between fears and perceived capability in SRA has important implications for training and suicide intervention more generally (30, 32). If training providers can identify participants who have greater SRA related fears prior to training, there may be scope to address the nature of their concerns during training. Notably, perceptions of having had sufficient training has been found to be related to higher levels of comfort in working with suicidal clients and having less fear in this work (30, 32). Therefore, identifying SRA related fears prior to training and seeking to address these may have positive effects on both fears and perceived capability. However, as such fears were only assessed at baseline in the current study, it is not possible to determine whether SRA related fears were reduced after STARS training.

### Changes in Competency After STARS Training and Predictors of Change

Consistent with Burnette et al.'s (22) model, we found overall positive impacts of STARS training on clinician competencies (attitudes, perceived capability, declarative knowledge) from pre- to post-training. The most pronounced changes following STARS training were evident for perceived capability and declarative knowledge, which is consistent with other SRA training findings (35, 56). We note, however, that due to the lack of control group such outcomes cannot conclusively be attributed to training.

In terms of predictors of change, we found that a lower amount of prior formal training and fewer years' experience predicted a greater increase in perceived capability at post-training, potentially due to their greater scope for improvement (i.e., those with less experience and training reported lower perceived capability at baseline). Consistent with the literature on attitudinal changes following SRA training (56, 57), we also found that participants had more positive attitudes toward suicide prevention after training. Further analysis showed that participants with less prior supervision/mentoring experienced the most change in attitudes. It is perhaps not surprising that those who engage in more supervision/mentoring around SRA tend to have positive attitudes prior to training, and therefore are less likely to demonstrate improvement following training.

Reluctance to intervene was not found to significantly change after training in either unadjusted or adjusted models. This contrasts with previous findings on levels of reluctance post GKT (44, 58). However, our finding is not surprising given the high scores (low reluctance) of participants at baseline, which may reflect a potential ceiling effect. Further, the results should be considered with caution considering the low internal reliability of this scale (Cronbach's α = 0.58) for our sample, and the original low to moderate alpha reported by the authors (α = 0.68) (44). Likewise, Ayer et al. (59) reported low internal consistency for this scale (e.g., α = 0.67 and α = 0.64 for Army and Marine Corps participants, respectively). Notably, reluctance to intervene has not previously been evaluated in SRA training (as opposed to GKT); thus, more research on the measurement of this construct and SRA training outcomes is recommended.

### Implications

This study provided evidence that three key theoretically based SRA competencies changed between pre- and post-STARS training, namely: more positive attitudes, greater perceived capability and increased declarative knowledge. Few training workshops with SRA competency-based frameworks supporting SPJ are currently available to Australian mental health workers. STARS training reflects such an approach and is well-aligned with Cramer et al.'s (15) SRA competencies, which we believe are critical for informing comprehensive evidence-informed knowledge and skills in SRA. There is a need for ongoing systematic evaluation of STARS, including a controlled trial of outcomes relative to other training (e.g., GKT).

Of note, our study highlighted the potential value of identifying professionals' fears around SRA practice at the outset of training due to the association with perceived capability. Facilitators may provide more emphasis on the impacts of multiple SRA related fears during training to increase participants' insight into impacts on their capability and suggest ways to address these (including seeking supervision). Further, it is possible that increased supervision around SRA practice increases positive attitudes toward suicide prevention, comfort and confidence in conducting SRA, which may subsequently enhance SRA interventions (60). Many benefits of supervision and mentoring have been identified for patient care and professionals' well-being in the mental health field generally (61, 62). However, scant attention has been paid to its application in regard to suicide-specific effective client outcomes, despite its proposed importance (60), and so this form of learning and support is strongly advocated.

### Strengths and Limitations

Our results should be interpreted in the context of some limitations. We used a convenience sample, which may not be representative of all mental health workers in Australia who work with suicidal clients. While we used a fairly homogenous sample of mental health workers in our study, in the sense that all had completed STARS training, as recommended by Jager et al. (63), our findings may not be generalized to other settings (e.g., emergency departments or acute inpatient units) where workers are repeatedly exposed to presentations of suicidality. Further, the majority of workshops were conducted in Queensland (9 out of 13), and therefore findings may not be generalizable to the entire Australian mental health workforce. Our self-selected sample of participants seeking SRA training could be particularly motivated to learn and potentially to have positive perceptions about their capability and attitudes toward working in this field. The low baseline levels of reluctance to intervene seem to support this view. We also did not include a control group for evaluating the efficacy of STARS training, and therefore cannot claim that changes in clinician competencies were due to the training *per se*.

The attrition analysis (35.1% drop-out) indicated that clinicians with more years of experience in suicide prevention (>10) were less likely to complete the post-training surveys. However, the impact of missing data on the analysis was managed by mixed linear modeling analyses. Due to the high turnover of the community mental health workforce in Australia (64), it was not possible to conduct a follow-up of the longer-term effects of training with the broader sample. We attempted to do follow-up contact with all participants on our STARS training database but received approximately 35% returned emails (returned email messages indicated “no longer at this address” or “email invalid”). Finally, the reluctance and attitude measures used in our study showed low internal consistency and therefore results on these measures should be interpreted with caution.

Our study has made a unique contribution to the suicide prevention training literature in that this is the first SRA training evaluated in Australia, which involves a semi-structured interview based on psycho-social needs assessment and structured professional judgement (SPJ) (8). Further, STARS protocol and training includes comprehensive safety planning based on Stanley and Brown's (20) safety planning intervention. The STARS training is underpinned by the globally proposed SRA competencies by Cramer et al. (15), and therefore is informed by empirical evidence about what is expected to be the minimum standards of competency for undertaking comprehensive, person-centered psycho-social risk assessment today.

## Conclusion

This study is the first to examine changes in clinician competencies in the context of STARS training. Our preliminary evaluation suggested that such training is associated with increased declarative knowledge, greater perceived capability and more positive attitudes toward suicide prevention. As a general finding, the participants most likely to benefit from STARS training had lower perceived capability or declarative knowledge about SRA prior to STARS or less prior supervision/mentoring experience. A long-term term follow-up of clinicians' use of and adherence to STARS protocol in practice is recommended in future research, along with a controlled evaluation of the impacts of STARS training relative to alternative training programs.

## Data Availability Statement

The raw data supporting the conclusions of this article can be made available upon a reasonable request.

## Ethics Statement

This study involving human participants was reviewed and approved by Griffith University Human Research Ethics Committee (Ref number: 2015/813/HREC). All participants provided their informed consent to participate in this study.

## Author Contributions

JH conceptualized and designed the study and coordinated the STARS training and evaluation project. TO, SS, DD, and EA are supervisors of Ph.D candidate JH. JH and KK analyzed data. JH wrote the manuscript. All authors contributed to the article and approved the submitted version.

## Conflict of Interest

The authors declare that the research was conducted in the absence of any commercial or financial relationships that could be construed as a potential conflict of interest.

## Publisher's Note

All claims expressed in this article are solely those of the authors and do not necessarily represent those of their affiliated organizations, or those of the publisher, the editors and the reviewers. Any product that may be evaluated in this article, or claim that may be made by its manufacturer, is not guaranteed or endorsed by the publisher.
