# Supplementary material for: Impact of Systematic Tailored Assessment for Responding to Suicidality (STARS) Protocol Training on Mental Health Professionals' Attitudes, Perceived Capabilities, Knowledge, and Reluctance to Intervene
Source: Front Psychiatry. 2022 Feb 8;12:827060. doi: 10.3389/fpsyt.2021.827060 (PMC8861433; doi:10.3389/fpsyt.2021.827060)
Supplement: Supplementary file 1 [file Data_Sheet_1.pdf]

Supplementary Material

Supplementary Material 1. STARS – Systematic Tailored Assessment for Responding to Suicidality

Systematic Tailored Assessment for Responding to Suicidality (STARS) protocol was first developed in 2015 by Hawgood and De Leo (Hawgood & De Leo, 2015) as an alternative to actuarial risk tools. It is a person-centered, psycho-social needs-based semi-structured interview (Hawgood & De Leo, 2016). STARS protocol includes empirically informed questions regarding indicators of suicidality (Part A), risk factors (Part B) and protective factors (Part C), to facilitate exploration of the clients' experience of suicidality and psycho-social factors. Instead of stratified suicide risk level outcomes however, STARS includes client-rated 'levels of concern' associated with elements of the suicidal enquiry, risk factors and protective factors. The client's narrative about what factors are most concerning to them and therapist judgement are integrated to determine priority foci for determining foreseeability, safety planning and immediate management responses. Finally, within the clinical notes section, the protocol provides for documentation of the client's suicidal state and psycho-social needs, and importantly, the commensurate actions proposed by the clinician for demonstration of standard of care, colleague consultation and follow-up and documentation of significant other involvement (where applicable) in safety planning (Stanley & Brown, 2012) (see STARS protocol structure below).

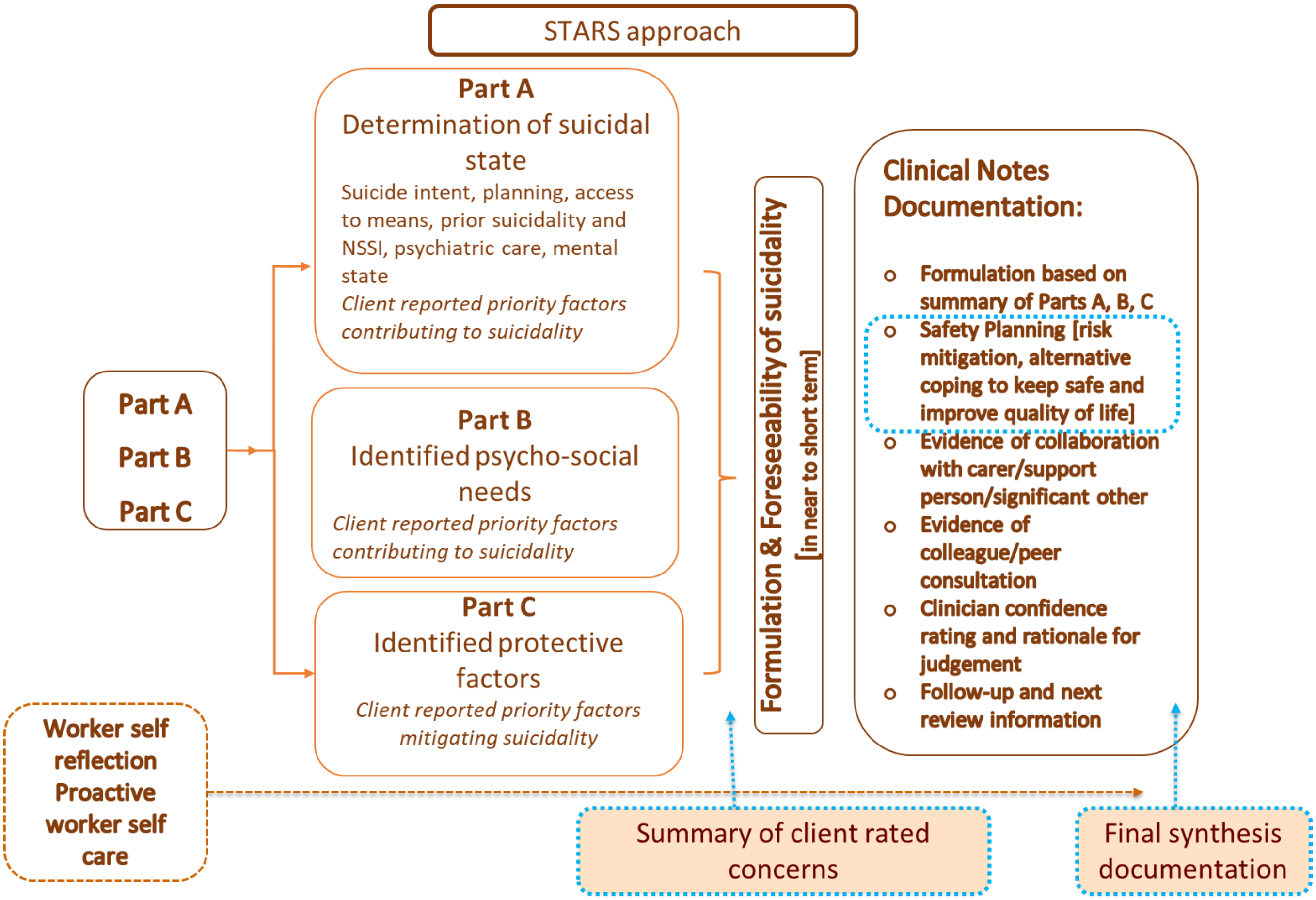
STARS approach
Part A
Determination of suicidal state
Suicide intent, planning, access to means, prior suicidality and NSSI, psychiatric care, mental state
Client reported priority factors contributing to suicidality
Part B
Identified psycho-social needs
Client reported priority factors contributing to suicidality
Part C
Identified protective factors
Client reported priority factors mitigating suicidality
Part A
Part B
Part C
Formulation & Foreseeability of suicidality
[in near to short term]
Clinical Notes Documentation:
Formulation based on summary of Parts A, B, C
Safety Planning [risk mitigation, alternative coping to keep safe and improve quality of life]
Evidence of collaboration with carer/support person/significant other
Evidence of colleague/peer consultation
Clinician confidence rating and rationale for judgement
Follow-up and next review information
Worker self reflection
Proactive worker self care
Summary of client rated concerns
Final synthesis documentation
